# Determination of dihydromyricetin in rat plasma by LC-MS/MS and its application to a pharmacokinetic study

**DOI:** 10.1080/13880209.2016.1266669

**Published:** 2016-12-12

**Authors:** Lu Liu, Xiaolan Yin, Xu Wang, Xiaohua Li

**Affiliations:** a Department of Endocrinology, Seventh People's Hospital of Shanghai University of TCM, Shanghai, China;; b Gamaknife Center, No 411 Hospital of the Chinese People's Liberation Army, Shanghai, China;; c Department of CT, First Affiliated Hospital of Nanyang Medical College, Nanyang, Henan, China

**Keywords:** Absorption, *Ampelopsis grossedentata*, bioavailability, flavonoid

## Abstract

**Context:** The pharmacokinetics properties of dihydromyricetin (DHM) are still unknown.

**Objective:** This study investigates the pharmacokinetic characteristics of DHM using a sensitive and reliable LC-MS/MS method.

**Materials and methods:** A rapid and sensitive LC-MS/MS method was developed for the determination of DHM in male Sprague–Dawley rat plasma. Twelve rats were equally randomized into two groups, including the intravenous group (2 mg/kg) and the oral group (20 mg/kg). Blood samples (250 μL) were collected at designated time points and analyzed using this method. The pharmacokinetic parameters were calculated using DAS 3.0 pharmacokinetic software.

**Results:** The calibration curve was linear within the range of 0.5–200 ng/mL (*r* > 0.998) with the lower limit of quantification at 0.5 ng/mL. After the intravenous injection, DHM reached a maximum concentration of 165.67 ± 16.35 ng/mL, and *t*_1/2_ was 2.05 ± 0.52 h. However, DHM was not readily absorbed and reached *C*_max_ 21.63 ± 3.62 ng/mL at approximately 2.67 h following the oral administration of DHM, and *t*_1/2_ was 3.70 ± 0.99 h. The MRT for the intravenous group and the oral group were 2.62 ± 0.36 and 5.98 ± 0.58 h, respectively. The *AUC*_(0-t)_ for the intravenous group and the oral group were 410.73 ± 78.12 and 164.97 ± 41.76 ng·L/mL, respectively, so the absolute bioavailability of DHM was 4.02% which was poor.

**Discussion and conclusion:** The results indicated that the bioavailability was poor. Further work needs to be conducted to investigate the reason for poor bioavailability and improve this situation.

## Introduction

*Ampelopsis grossedentata* (Hand.-Mazz.) W. T. Wang is a medicinal and edible plant that is widely distributed in southern China, and its tender stems and leaves have been used as medicinal tea for the prevention and treatment of common cold, sore throat, and icteric viral hepatitis for hundreds of years (Hou et al. 2015; Bi et al. 2016; Chen et al. 2016). Dihydromyricetin[(2*R*,3*R*)-3,5,7-trihydroxy-2-(3,4,5-trihydroxyphenyl)-2,3-dihydrochromen-4-one, PubChem CID: 161557] (DHM) is the most abundant and active flavonoid component isolated from *Ampelopsis grossedentata* (Du et al. 2002; Xia et al. 2014; Zhong et al. 2014). DHM possesses numerous biological and pharmacological activities, including antioxidative, antiinflammatory, hepatoprotective, lipid and blood glucose regulatory, and anticancer effects (Huang et al. 2013; Chen et al. 2015; Hou et al. 2015; Ji et al. 2015; Jiang et al. 2014, 2015; Meng et al. 2015). Li (2006) has found that DHM could reduce the serum level of total cholesterol and triglycerides while increasing the serum high-density lipoprotein cholesterol level in hyperlipidemia rats after oral administration. Qin et al. (2001) have also found that DHM could reduce the blood glucose level in alloxan-induced diabetic or hyperglycemic mice. Zou et al. (2014) have found that DHM could improve physical performance under simulated high altitude conditions by preserving mitochondrial function of skeletal muscles and ameliorate insulin resistance in skeletal muscles by regulating autophagy both *in vitro* and *in vivo*.

Because of the potent pharmacological activities of DHM, it is of great significance to investigate the pharmacokinetic properties. Zhang et al. (2007) have investigated the pharmacokinetic profiles of DHM after oral administration of *Ampelopsis grossedentata* decoction using a simple and sensitive HPLC-DAD method. Tong et al. (2015) have developed a sensitive and reliable LC-MS/MS method to determine the concentration of DHM after its oral administration at a dose of 100 mg/kg, and the results indicated that DHM was poorly absorbed into blood. However, to the best of our knowledge, there are little data available for defining the oral bioavailability of DHM. To enhance the development potential of DHM, it is urgent to investigate the pharmacokinetic profiles of DHM, especially its bioavailability characteristics.

This study investigates the pharmacokinetic characteristics following oral or intravenous administration of DHM by a sensitive and reliable LC-MS/MS method.

## Materials and methods

### Chemicals and reagents

**Figure 1.**
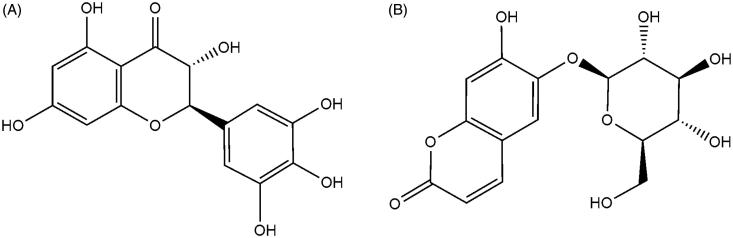
The chemical structures of DHM (A) and esculin (B).

DHM (purity >98%) and esculin (purity >98%) were purchased from the National Institute for the Control of Pharmaceutical and Biological Products (Beijing, China), and their structures are shown in Figure 1. Acetonitrile and methanol were purchased from Fisher Scientific (Fair Lawn, NJ). Formic acid was purchased from Anaqua Chemicals Supply Inc. Limited (Houston, TX). All the other chemicals were of analytical grade or better.

### Instrumentation and conditions

The analysis was performed on an Agilent 1290 series liquid chromatography system (Agilent Technologies, Palo Alto, CA) and an Agilent 6470 triple-quadruple mass spectrometer (Agilent Technologies, CA). Chromatographic separation of DHM and esculin was performed on Waters X-bridge C_18_ column (1.0 × 50 mm, 2.5 μm, USA) at 35 °C. The mobile phase was water (0.1% formic acid) and acetonitrile (15:85, v:v) at a flow rate of 0.4 mL/min.

The mass scan mode was negative MRM mode. The precursor ion and product ion are *m/z* 319.0 → 193.0 for DHM, and *m/z* 339.0 → 176.9 for esculin, respectively. The collision energy for DHM and IS were 25, and 20 ev, respectively. The MS/MS conditions were optimized as follows: fragmentor, 120 V; capillary voltage, 3.5 kV; nozzle voltage, 500 V; nebulizer gas pressure (N_2_), 40 psig; drying gas flow (N_2_), 10 L/min; gas temperature, 350 °C; sheath gas temperature, 400 °C; sheath gas flow, 10 L/min.

### Animals

Male Sprague–Dawley (SD) rats weighing 220–250 g were provided by the Experimental Animal Center of the Shanghai University of Traditional Chinese Medicine (Shanghai, China). Rats were bred in a breeding room at 25 °C, 60 ± 5% humidity, and a 12 h dark-light cycle. Tap water and normal chow were given *ad libitum*. All the experimental animals were housed under the above conditions for a 7 day acclimation, and were fasted overnight before the experiments.

### *In vivo* pharmacokinetic study

The animal facilities and protocols were approved by the Institutional Animal Care and Use Committee. All procedures were in accordance with the National Institute of Health guidelines regarding the principles of animal care. Rats were fasted for 12 h with free access to water prior to the pharmacokinetic study. Twelve rats were equally randomized to two groups and were treated as followings: intravenous injection of DHM in saline was administrated through lateral tail vein at a dose of 2 mg/kg. An oral gavage of DHM dispersed in oral suspension vehicle was given to rats at a dose of 20 mg/kg. Blood samples (250 μL) were collected into a heparinized tube via the oculi chorioideae vein at 0.083, 0.167, 0.33, 0.5, 1, 2, 4, 6, 8, 12, and 24 h, respectively. After centrifugation at 5000 rpm for 10 min, plasma samples were obtained and frozen at −40 °C until analysis.

### Plasma sample preparation

Plasma sample (100 μL) was spiked with 10 μL of the esculin (100 ng/mL), and then the mixture was extracted with 190 μL of acetonitrile by vortexing for 1 min. After centrifugation at 12,500 rpm for 10 min, the supernatant of 5 μL was injected into the LC-MS/MS for the determination.

### Preparation of standard and quality control samples

A stock solution of DHM was prepared in methanol at a concentration of 5 mg/mL. The stock solution of esculin was prepared in acetonitrile at a concentration of 2 mg/mL. Calibration standard samples for DHM were prepared in blank rat plasma at concentrations of 0.5, 1, 2, 5, 10, 20, 50, 100, and 200 ng/mL. The quality control (QC) samples were prepared at low (1 ng/mL), medium (20 ng/mL), and high (160 ng/mL) concentrations in the same way as the plasma samples for calibration, and QC samples were stored at −40 °C until analysis.

## Method validation

The method validation assay was performed according to the United States Food and Drug Administration (FDA) guidelines. Selectivity was investigated by comparing the chromatograms of six different batches of blank rat plasma with the corresponding spiked plasma to monitor interference of endogenous substances and metabolites. To obtain the calibration curve, seven concentrations of the calibration standard were processed and determined as described above. The linearity of calibration curves was constructed by plotting peak area ratios (*y*) of the analytes to IS against the nominal concentration (*x*) of analytes with weighted (1/*x*^2^) least square linear regression. The lower limit of detection (LLOD) and lower limit of quantification (LLOQ) were determined as the concentration of the analyte with a signal-to-noise ratio at 3 and 10, respectively. The intra-day precision and accuracy of the method were confirmed by determining QC samples at three different concentrations five times on a single day, and the inter-day precision and accuracy were assessed by determining the QC samples over three consecutive days. For each concentration, five replicates were prepared. Relative standard deviation (RSD) and relative error (RE) were used to express the precision and accuracy, respectively.

The extraction recovery was assessed by comparing peak areas obtained from extracted spiked samples with those originally spiked in the blank plasma samples. The matrix effect was evaluated by comparing the peak areas of the post-extracted spiked QC samples with those of corresponding standard solutions. These procedures were repeated for five replicates at three QC concentration levels. For sample stability, three levels of QC samples were determined under different conditions, including short-term stability at room temperature for 24 h, long-term stability at −40 °C for 30 days, and three freeze-thaw cycles at −40 °C.

### Data analysis

The pharmacokinetic parameters, including area under the plasma concentration-time curve (*AUC*), maximal plasma concentration (*C*_max_), the time for maximal plasma concentration (*T*_max_), and mean residence time (*MRT*) were calculated using DAS 3.0 pharmacokinetic software (Chinese Pharmacological Association, Anhui, China).

## Results and discussion

### Sample preparation

Due to the complex nature of plasma, a sample pretreatment procedure is needed to remove protein and potential interferences before LC-MS/MS analysis. In this study, protein precipitation, solid phase extraction and liquid–liquid extraction were investigated to achieve good resolution and high recovery of analytes from spiked biologic matrices. Finally, direct protein precipitation method using acetonitrile was selected for biological sample preparation.

### Chromatography and mass spectrometry

**Figure 2.**
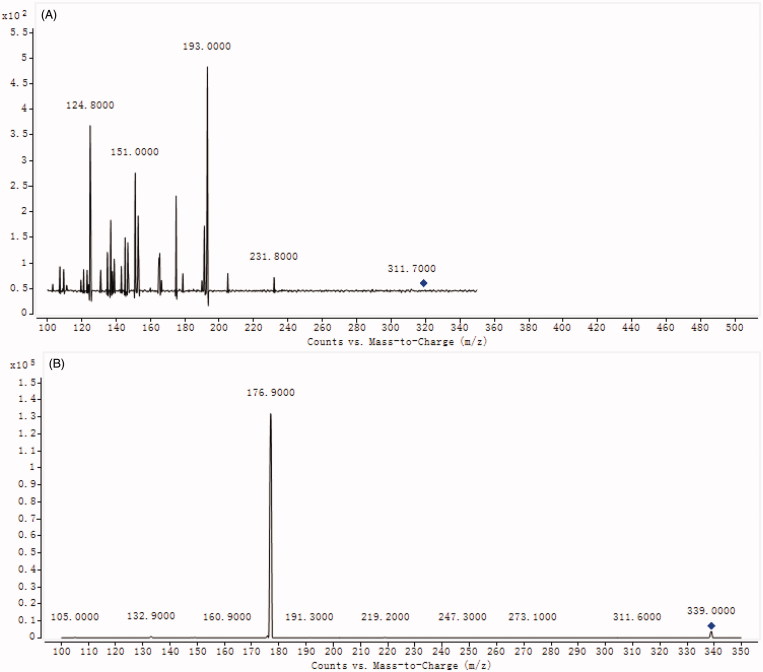
The mass spectra of DHM (A) and esculin (B).

**Figure 3.**
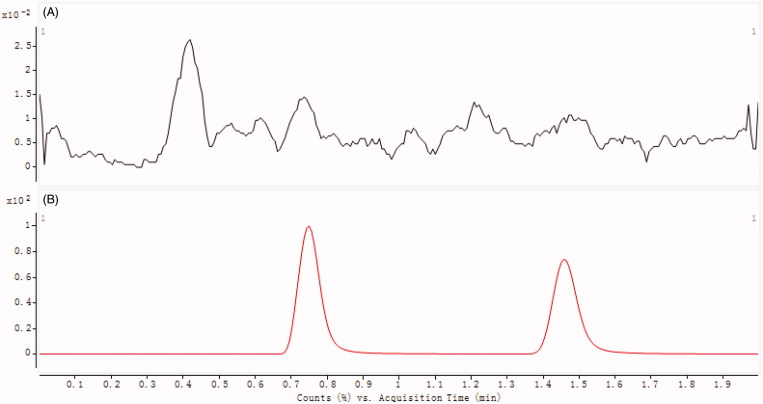
Chromatograms of blank plasma (A), and plasma spiked with DHM and esculin (B). B1: esculin; B2: DHM.

As shown in Figure 2, the optimized mass transition ion-pairs for quantification, including precursor and product ions, were *m/z* 319.0 → 193.0 for DHM and *m/z* 339.0 → 176.9 for esculin, respectively. Blank plasma, plasma spiked with DHM and esculin are shown in Figure 3. No significant interference substances were observed at the retention time of DHM (0.75 min) of IS (1.47 min) in plasma samples.

### Method validation

The standard curve for DHM in plasma was linear in the concentration range of 0.5–200 ng/mL (*y* = 0.125*x* + 0.116, *r* = 0.999). The LLOQ and LLOD were 0.5 and 0.18 ng/mL, respectively.

Intra-day and inter-day precision and accuracy were determined by measuring six replicates of QC samples at three concentration levels in rat plasma. The precision and accuracy data are shown in Table 1. These results demonstrated that the precision and accuracy values were well within an acceptable range of 15%.

**Table 1.** The intra-day and inter-day precision and accuracy of DHM in plasma samples.

Analyte	Plasma samples(ng/mL)	Intra-day			Inter-day		
		Concentrationmeasured (ng/mL)	Precision(%, RSD)	Accuracy(%, RE)	Concentrationmeasured (ng/mL)	Precision(%, RSD)	Accuracy(%, RE)
DHM	1	0.93	6.68	−7.00	1.08	7.36	8.00
	20	21.36	7.25	6.80	18.75	5.59	−6.25
	160	146.98	8.16	−8.14	171.25	6.87	7.03
Esculin	100	105.25	5.62	5.25	107.62	6.35	7.62

The mean extraction recoveries determined using three replicates of QC samples at three concentration levels in rat plasma were 88.36 ± 6.23, 92.55 ± 5.36, and 91.05 ± 6.35% for 1, 20 and 160 ng/mL, respectively.

For ionization, the peak areas of DHM after spiking evaporated plasma samples at three concentration levels were comparable to those of similarly prepared aqueous standard solutions (ranging from 93.54 to 102.68%), suggesting that there was no measurable matrix effect that interfered with DHM determination in rat plasma.

The stability of DHM in plasma was evaluated by analyzing three replicates of quality control samples containing 1, 20 and 160 ng/mL DHM after short-term storage (25 °C, 24 h), long-term cold storage (−40 °C, 30 days) and within three freeze (−40 °C)-thaw (room temperature) cycles. As shown in Table 2, all of the samples displayed 90–110% recoveries after various stability tests. Taken together, the above results show that a rapid, simple and sensitive method for analyzing DHM in rat plasma samples.

**Table 2.** Stability of DHM in plasma samples (*n* = 3).

Analyte	Plasma samples(ng/mL)	Stability (%, RE)		
		Short-term(24 h at room temperature)	Long-term(30 days at −40 °C)	Three freeze(−40 °C) – thaw(room temperature)cycles
DHM	1	5.36	7.35	5.58
	20	7.25	6.98	7.26
	160	6.15	8.16	8.16

### Pharmacokinetic study

**Figure 4.**
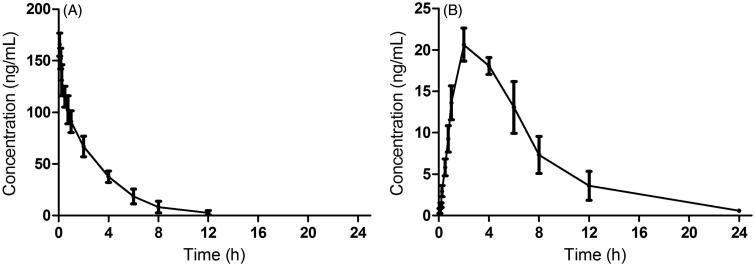
The pharmacokinetic profiles of DHM in rats after intravenous administration of DHM (A) at dosage of 2 mg/kg and oral administration of DHM (B) at 20 mg/kg.

The validated analytical method was employed to study the pharmacokinetic behaviours of DHM in rats. The mean plasma concentration-time curves of DHM after both the intravenous and the oral administration of DHM are presented in Figure 4.

The pharmacokinetic parameters were calculated using the noncompartmental method with DAS 3.0 pharmacokinetic software (Chinese Pharmacological Association, Anhui, China). The pharmacokinetic parameters are shown in Table 3. The oral bioavailability was calculated by using *AUC_oral/dose_* divided by *AUC_iv/dose_*.

**Table 3.** Pharmacokinetic parameters of DHM in rats after intravenous (2 mg/kg) or oral administration (20 mg/kg) of DHM (*n* = 6, Mean ± SD).

Parameter	Intravenous	Oral
*T*_max_ (h)	–	2.67 ± 1.25
*C*_max_ (ng/mL)	165.67 ± 16.35	21.63 ± 3.62
*t*_1/2_ (h)	2.05 ± 0.52	3.70 ± 0.99
*AUC*_(0-t)_ (ng·h/mL)	410.73 ± 78.12	164.97 ± 41.76
*AUMC*_(0-t)_ (ng·h^2^/mL)	1091.89 ± 353.98	997.26 ± 256.87
*MRT* (h)	2.62 ± 0.36	5.98 ± 0.58

After 2 mg/kg intravenous injection, the concentration of DHM reached a maximum of 165.67 ± 16.35 ng/mL, and *t*_1/2_ was 2.05 ± 0.52 h. However, after the oral administration of 20 mg/kg DHM, DHM was not readily absorbed and reached *C*_max_ 21.63 ± 3.62 ng/mL at approximately 2.67 h, and *t*_1/2_ was 3.70 ± 0.99 h. These results indicated DHM was poorly absorbed and slowly eliminated. The MRT for the intravenous group and the oral group were 2.62 ± 0.36 and 5.98 ± 0.58 h, respectively. The *AUC*_(0-t)_ for the intravenous group and the oral group was 410.73 ± 78.12 and 164.97 ± 41.76 ng·L/mL, respectively. Thus, the absolute bioavailability of DHM by oral route was 4.02%, and the results indicated that the bioavailability of DHM was poor. Tong et al. (2015) also reported that the absorption of DHM was poor after oral administration of DHM. Further studies need to be conducted to investigate the reason for its poor absorption and further measures to improve its bioavailability are needed. Hu (2007) have indicated that poor absorption is the major reason for the poor bioavailability of ﬂavonoids, therefore, how to improve the poor oral bioavailability of DHM is a huge obstacle for pharmacological investigations of DHM or ﬂavonoids. Wang et al. (2016) have reported that oral bioavailability of DHM could be improved though inhibiting precipitation of soluble cocrystals by a crystallization inhibitor, and their results also showed that cocrystallization can be used to improve physiochemical and biopharmaceutic properties of biopharmaceutics classification system IV drugs.

## Conclusions

In conclusion, a rapid, simple and sensitive LC-MS/MS method has been developed and successfully applied to determine the concentration of DHM in rat plasma. With the application of this method, the pharmacokinetic characteristics of DHM in rats were investigated. The results indicated that the oral bioavailability of DHM was poor. We hope this study will be helpful for further pharmacological research, and more research is needed to investigate the reason for poor bioavailability so improvements can be made.
